# Oxidative Stress Parameters in the Liver of Growing Male Rats Receiving Various Alcoholic Beverages

**DOI:** 10.3390/nu12010158

**Published:** 2020-01-06

**Authors:** Aleksandra Kołota, Dominika Głąbska, Michał Oczkowski, Joanna Gromadzka-Ostrowska

**Affiliations:** Department of Dietetics, Institute of Human Nutrition Sciences, Warsaw University of Life Sciences (SGGW-WULS), 159c Nowoursynowska Street, 02-776 Warsaw, Poland; dominika_glabska@sggw.pl (D.G.); michal_oczkowski@sggw.pl (M.O.); joanna_gromadzka_ostrowska@sggw.pl (J.G.-O.)

**Keywords:** ethanol, beer, red wine, rats, adolescent, liver, ethanol metabolism, inflammation, oxidative stress, liver enzymes

## Abstract

Typical alcohol consumption begins in the adolescence period, increasing the risk of alcoholic liver disease (ALD) in adolescents and young adults, and while the pathophysiology of ALD is still not completely understood, it is believed that oxidative stress may be the major contributor that initiates and promotes the progression of liver damage. The aim of the present study was to assess the influence of alcohol consumption on the markers of oxidative stress and liver inflammation in the animal model of prolonged alcohol consumption in adolescents using various alcoholic beverages. In a homogenic group of 24 male Wistar rats (4 groups—6 animals per group), since 30th day of life, in order to mimic the alcohol consumption since adolescence, animals received (1) no alcoholic beverage (control group), (2) ethanol solution, (3) red wine, or (4) beer (experimental groups) for 6 weeks. Afterwards, the activities of alcohol dehydrogenase (ADH), alanine aminotransferase (ALT), and aspartate aminotransferase (AST), as well as levels of cytochrome P450-2E1 (CYP2E1), thiobarbituric acid-reactive substances (TBARS), protein carbonyl groups, tumor necrosis factor-α (TNF-α), and interleukine-10 (IL-10) were measured in liver homogenates. The difference between studied groups was observed for CYP2E1 and protein carbonyl groups levels (increased levels for animals receiving beer compared with control group), as well as for ALT activity (decreased activity for animals receiving beer compared with other experimental groups) (*p* < 0.05). The results suggested that some components of beer, other than ethanol, are responsible for its influence on the markers of oxidative stress and liver inflammation observed in the animal model of prolonged alcohol consumption in adolescents. Taking this into account, beer consumption in adolescents, which is a serious public health issue, should be assessed in further studies to broaden the knowledge of the progression of liver damage caused by alcohol consumption in this group.

## 1. Introduction

According to the data of the World Health Organization (WHO) [1], one in four adolescents aged 15–19 years declares the consumption of alcoholic beverages during the previous month, while the highest number of current drinkers is recorded in European countries. Moreover, alcohol abuse accounts for around 13.5% of deaths among adolescents, indicating that it is currently becoming a serious social and health issue [1,2,3].

Typical alcohol consumption begins in the adolescence period and is observed until adulthood [4], which increases risk of liver diseases in adults and elderly people, as described in the studies of Bosetti et al. [5] and Hagström et al. [6]. Excessive intake of alcohol is the main reason for liver diseases, including cirrhosis and cancer, as well as acute and chronic failure [7], while the most common health-related consequences include alcoholic liver disease (ALD), which is a condition associated with various morphological changes in the liver, ranging from steatosis alone to advanced steatosis accompanied by inflammation, fibrosis, and/or cirrhosis [8].

Recently, the prevalence of ALD in adolescents and young adults is found to be rapidly increasing [9], and in some countries, for example China, it has doubled over the last 10 years [10]. Furthermore, the mortality rate related to chronic liver diseases that occur due to the excessive consumption of alcohol has also been increasing, especially among young adults aged 25–34 years, as observed by a study in Great Britain [11].

The pathophysiology of ALD is still not completely understood, but it is believed that oxidative stress may be the major contributor [12] that initiates and promotes the progression of liver damage [13]. Prolonged consumption of alcohol induces the microsomal ethanol-oxidizing system (MEOS), intensifying the process of transformation of ethanol to acetaldehyde, which damages various organs, mainly the liver. MEOS induction also increases the generation of reactive oxygen species (ROS), which not only leads to the damage of hepatocytes but also modifies the gut microbiota into those stimulating the generation of endotoxins and consequently, participates in liver damage [14]. As indicated above, some authors have also emphasized that the influence of alcohol on gut microbiota plays an important role in ALD development [15].

ALD is associated with alcoholic fatty liver disease, observed in almost all individuals as a result of chronic alcohol abuse, while liver inflammation and fibrosis are stated for 10–35% of such individuals, and cirrhosis for 10–15%. For adults, fatty liver disease and inflammation may be reversed due to abstinence, but serious ALD results in bad prognosis [7]. For adolescents, there are no data presenting ALD development, but taking into account the increase of alcohol consumption in this group [16], this problem is becoming a vital public health issue.

In the research conducted in the models of chronic alcohol abuse in adults, the significant influence of alcohol consumption on the markers of oxidative stress and liver inflammation was proven [17,18], but such an effect was not studied so far in developing organisms, as well as for the alcoholic beverages other than ethanol. As such studies analyzing these factors should be conducted in the animal models of alcohol abuse in adolescents, and not in human subjects, due to ethical issues, the aim of the present study was to assess the influence of alcohol consumption on the markers of oxidative stress and liver inflammation in the animal model of prolonged alcohol consumption in adolescents using various alcoholic beverages.

## 2. Materials and Methods

All the study protocols were approved by the 3rd Local Ethics Committee for Animal Experiments at the Warsaw University of Life Sciences, Poland (26/2007).

### 2.1. Characteristics of the Studied Animals

The study was conducted in a homogenic group of 24 male Wistar rats of a Wistar Cmd: WI(WU) strain, of an initial body mass of 93.5 ± 1.29 g that were provided by the Mossakowski Medical Research Centre of the Polish Academy of Sciences (Warsaw, Poland). During the whole experimental period animals were housed individually, while the temperature of 23 ± 2 °C, relative humidity of 50–60%, and 12/12 h light–dark cycle were applied.

After the age of 20 days, each animal was attributed to one of the four groups: (1) control group receiving no alcoholic beverage, (2) experimental group receiving ethanol solution, (3) experimental group receiving red wine, (4) experimental group receiving beer. Afterwards, until the 30th day of life, animals were subjected to acclimatization procedure, while animals from the experimental groups were receiving the solution of applicable alcoholic beverage (ethanol, beer, or red wine) daily. Every second day the amount of alcohol in the provided water solution was increasing, and for each experimental group, the ethanol solution was identical (2%, 4%, 6%, 8%, and 10% of ethanol solution). The solutions were prepared while using absolute ethyl alcohol (Chempur, Piekary Śląskie, Poland) (for a group receiving ethanol solution), dry red wine (Sophia Sakar, Cabernet Sauvignon, Domain Menada, Stara Zagora city, Bulgaria, 11% alc. vol.) (for a group receiving red wine), and beer (Faxe Extra Strong, Royal Unibrew, Faxe, Denmark, 10% alc. vol.) (for a group receiving beer), as well as tap water to dilute the alcoholic beverage to obtain an adequate solution. From the 30th day of life for the 6 weeks, animals from the experimental groups received daily the alcoholic beverages characterized by 10% ethanol solution (Table 1).

During the acclimatization period, as well as during the whole experimental period, animals received ad libitum a standard laboratory diet (Wytwórnia Pasz Morawski, Kcynia, Poland), while the detailed nutritional value was presented in the previous study [19]. During the whole period, animals also had free access to pure water.

The presented study was conducted in a male Wistar rats model, while the alcohol consumption was introduced from the 30th day of life in order to mimic the alcohol consumption since adolescence, as for rats, this day is attributed to early youth [20]. At the same time, the study was conducted for 6 weeks, in order to mimic the alcohol consumption until adulthood [21].

### 2.2. Experimental Procedures

After the acclimatization period, animals from the experimental groups amounted the 10% of ethanol in the solution of alcoholic beverage that they were receiving, and it was the final experimental solution that was applied during the whole experimental period. Similarly, as during the acclimatization period, animals were receiving the solution of applicable alcoholic beverage (ethanol, beer, or red wine) daily. The solutions were prepared similarly, as during the acclimatization period, while absolute ethyl alcohol (Chempur, Piekary Śląskie, Poland) (for a group receiving ethanol solution) or dry red wine (Sophia Sakar, Cabernet Sauvignon, Domain Menada, Stara Zagora city, Bulgaria, 11% alc. vol.) (for a group receiving red wine) were diluted using tap water to obtain 10% of ethanol in the solution of alcoholic beverage, and beer (Faxe Extra Strong, Royal Unibrew, Faxe, Denmark, 10% alc. vol.) (for a group receiving beer) was applied with no dilution.

The experimental period was planned for 6 weeks. During the experimental period, animal from the experimental groups were receiving applicable alcoholic beverages ad libitum and no water during the dark cycle, but during the light cycle, they received applicable alcoholic beverages ad libitum and at the same time, also water ad libitum, which is the commonly applied procedure to force alcohol consumption in the animal model [22]. At the same time, animals from the control group were receiving water ad libitum and no alcoholic beverages, during both dark and light cycle.

The intake of alcoholic beverage solution was controlled daily, as well as the intake of diet. At the same time, the body mass of animals was controlled weekly.

### 2.3. Analytical Methods and Measurements

The activity of alcohol dehydrogenase (ADH) was measured in liver homogenates by a spectrophotometric method, while the #K787-100 Alcohol Dehydrogenase Activity Assay kit (BioVision, Milpitas, CA, USA) was used, and the result of ADH activity was expressed as mU per mg of protein.

The level of cytochrome P450-2E1 (CYP2E1) was measured in liver homogenates by an immunoenzymatic method, while the SEA988Ra ELISA kit for Rat Cytochrome P450-2E1 (USCN Life Science Inc. Wuhan, China) was used, and the result of CYP2E1 level was expressed as mU per mg of protein.

The level of thiobarbituric acid-reactive substances (TBARS) was measured in liver homogenates by a spectrophotometric method of Ohkawa et al. [23], and the result of TBARS level was expressed as µmol per mg of protein.

The level of protein carbonyl groups was measured in liver homogenates by a colorimetric method by Levine et al. [24], after samples preparation according to the procedure by Reznick and Packer [25], and the result of protein carbonyl group level was expressed as nmol per mL.

The level of tumor necrosis factor-α (TNF-α) was measured in liver homogenates by a immunoenzymatic method, while the Quantikine Rat TNF-α RTA00 kit (R&D Systems, Inc., Minneapolis, MN, USA) was used, and the result of TNF-α level was expressed as pg per mg of protein.

The level of interleukine-10 (IL-10) was measured in liver homogenates by an immunoenzymatic method, while the Quantikine Rat IL-10 R1000 kit (R&D Systems, Inc., Minneapolis, MN, USA) was used, and the result of IL-10 level was expressed as pg per mg of protein.

The activity of alanine aminotransferase (ALT) was measured in liver homogenates by a spectrophotometric method, while the ALT/GPT Liquid kit (P.T.H. Hydrex, Warsaw, Poland) was used, and the result of ALT activity was expressed as U per mg of protein.

The activity of aspartate aminotransferase (AST) was measured in liver homogenates by a spectrophotometric method, while the AST/GOT Liquid kit (P.T.H. Hydrex, Warsaw, Poland) was used, and the result of AST activity was expressed as U per mg of protein.

The level of protein was measured in liver homogenates by a spectrophotometric method of Bradford [26], while using 110 306 Bioquant^®^ Protein (Merck, Darmstadt, Germany) and V900933 bovine serum albumin (Sigma-Aldrich, St. Louis, MO, USA), as a standard.

### 2.4. Statistical Analysis

The normality of distribution was verified by using the Shapiro–Wilk test, and the parametric distribution for all the variables was confirmed. The values observed for groups were compared by using the one-way analysis of variance (ANOVA) accompanied by the Duncan post hoc multiple comparison test. While concluding, *p* ≤ 0.05 was interpreted as significant. All statistical analyses were performed by using the Statistica software version 13.1 (StatSoft Inc., Tulsa, OK, USA).

## 3. Results

The results of body mass and alcohol consumption for compared groups were presented in the previous study [19].

**Figure 1:**
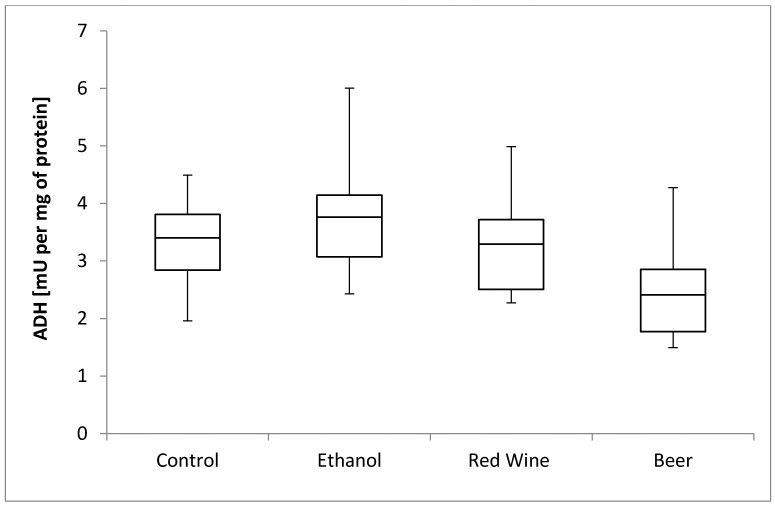
The box plots of the ADH (alcohol dehydrogenase) activity (mU per mg of protein) assessed in rat liver homogenates after 6 weeks of experiment, stratified by the alcoholic beverage.

The box plots of the ADH activity (mU per mg of protein) assessed in liver homogenates after 6 weeks of experiment, stratified by the alcoholic beverage are presented in Figure 1. It was stated that there is no statistically significant influence of receiving alcoholic beverage by animals on ADH activity, as well as no differences between experimental groups receiving various alcoholic beverages (*p* = 0.2227).

**Figure 2:**
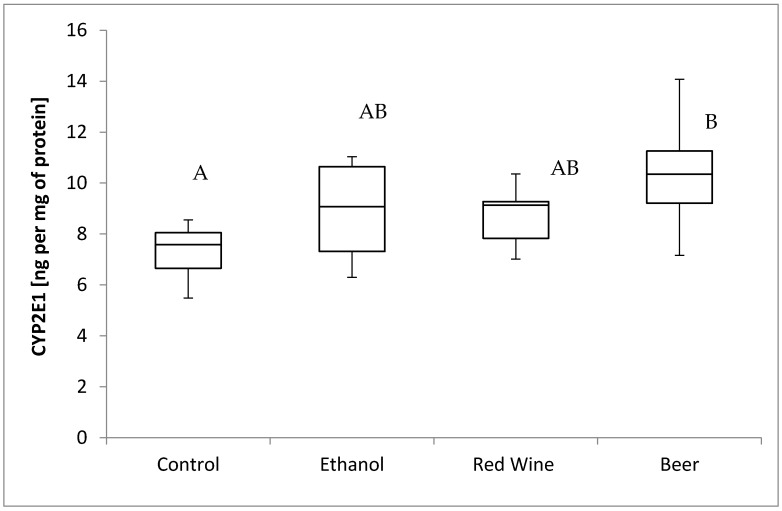
The box plots of the CYP2E1 (cytochrome P450-2E1) level (ng per mg of protein) assessed in rat liver homogenates after 6 weeks of experiment, stratified by the alcoholic beverage (different letters are attributed to groups differing significantly, *p* < 0.05).

The box plots of the CYP2E1 level (ng per mg of protein) assessed in liver homogenates after 6 weeks of experiment, stratified by the alcoholic beverage are presented in Figure 2. It was stated that there is a close to significance influence of receiving alcoholic beverage by animals on CYP2E1 level (*p* = 0.0536). It was confirmed for compared results obtained for a control group and experimental group receiving beer, as animals receiving beer were characterized by significantly higher results of CYP2E1 level in multiple comparisons than those from other groups (*p* < 0.05).

**Figure 3:**
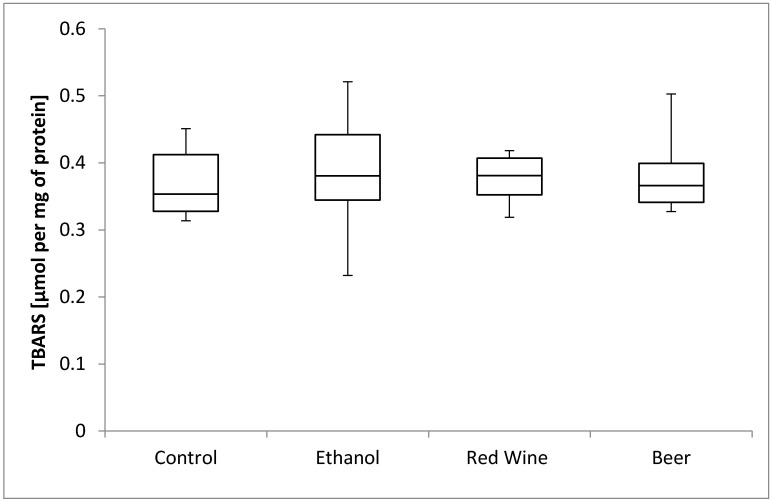
The box plots of the TBARS (thiobarbituric acid-reactive substances) level (μmol per mg of protein) assessed in rat liver homogenates after 6 weeks of experiment, stratified by the alcoholic beverage.

The box plots of the TBARS level (μmol per mg of protein) assessed in liver homogenates after 6 weeks of experiment, stratified by the alcoholic beverage are presented in Figure 3. It was stated that there is no statistically significant influence of receiving alcoholic beverage by animals on TBARS level, as well as no differences between experimental groups receiving various alcoholic beverages (*p* = 0.9799).

**Figure 4:**
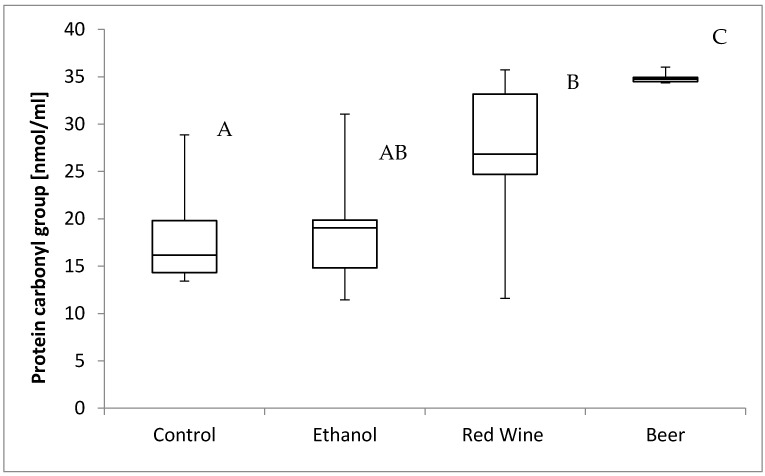
The box plots of the protein carbonyl group level (nmol per mL) assessed in rat liver homogenates after 6 weeks of experiment, stratified by the alcoholic beverage (different letters are attributed to groups differing significantly, *p* < 0.05).

The box plots of the protein carbonyl group level (nmol per mL) assessed in liver homogenates after 6 weeks of experiment, stratified by the alcoholic beverage are presented in Figure 4. It was stated that there is a statistically significant influence of receiving alcoholic beverage by animals on protein carbonyl group level (*p* = 0.0013). It was confirmed for compared results obtained for a control group and experimental groups, as animals receiving beer were characterized by significantly higher results of protein carbonyl group level in multiple comparisons than those from other groups, and animals receiving red wine were characterized by a significantly higher results than observed for control group (*p* < 0.05).

**Figure 5:**
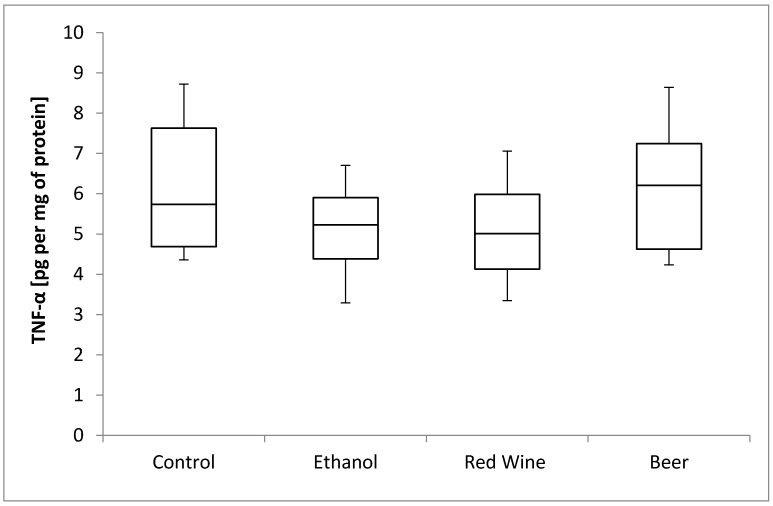
The box plots of the TNF-α (tumor necrosis factor-α) level (pg per mg of protein) assessed in rat liver homogenates after 6 weeks of experiment, stratified by the alcoholic beverage.

The box plots of the TNF-α level (pg per mg of protein) assessed in liver homogenates after 6 weeks of experiment, stratified by the alcoholic beverage are presented in Figure 5. It was stated that there is no statistically significant influence of receiving alcoholic beverage by animals on TNF-α level, as well as no differences between experimental groups receiving various alcoholic beverages (*p* = 0.4531).

**Figure 6:**
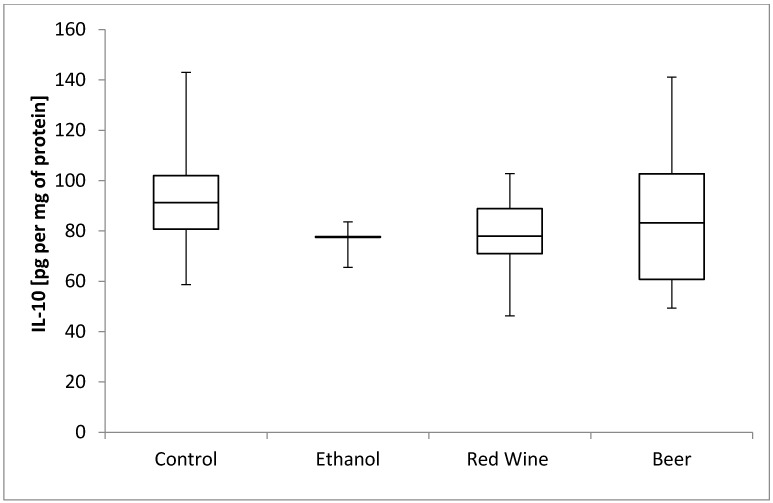
The box plots of the IL-10 (interleukine-10) level (pg per mg of protein) assessed in rat liver homogenates after 6 weeks of experiment, stratified by the alcoholic beverage.

The box plots of the IL-10 level (pg per mg of protein) assessed in liver homogenates after 6 weeks of experiment, stratified by the alcoholic beverage are presented in Figure 6. It was stated that there is no statistically significant influence of receiving alcoholic beverage by animals on IL-10 level, as well as no differences between experimental groups receiving various alcoholic beverages (*p* = 0.5921).

**Figure 7:**
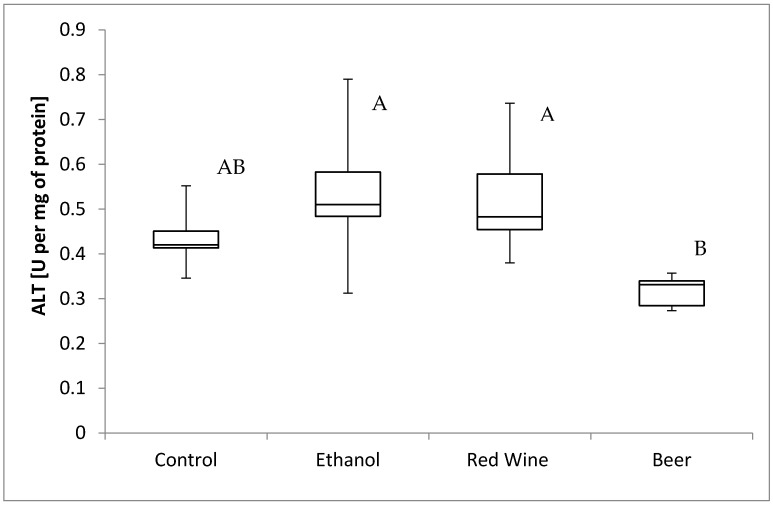
The box plots of the ALT (alanine aminotransferase) activity (U per mg of protein) assessed in rat liver homogenates after 6 weeks of experiment, stratified by the alcoholic beverage (different letters are attributed to groups differing significantly, *p* < 0.05).

The box plots of the ALT activity (U per mg of protein) assessed in liver homogenates after 6 weeks of experiment, stratified by the alcoholic beverage are presented in Figure 7. It was stated that there is a statistically significant influence of receiving alcoholic beverage by animals on alanine aminotransferase activity (*p* = 0.0162). It was confirmed for compared results obtained for experimental groups, as animals receiving beer were characterized by significantly lower results of alanine aminotransferase activity in multiple comparisons than those from groups receiving wine and ethanol (*p* < 0.05).

**Figure 8:**
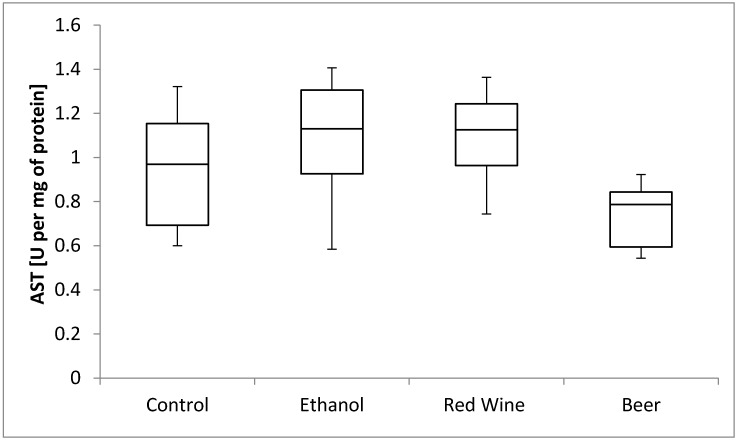
The box plots of the AST (aspartate aminotransferase) activity (U per mg of protein) assessed in rat liver homogenates after 6 weeks of experiment, stratified by the alcoholic beverage.

The box plots of the AST activity (U per mg of protein) assessed in liver homogenates after 6 weeks of experiment, stratified by the alcoholic beverage are presented in Figure 8. It was stated that there is no statistically significant influence of receiving alcoholic beverage by animals on aspartate aminotransferase activity, as well as no differences between experimental groups receiving various alcoholic beverages (*p* = 0.1282).

## 4. Discussion

The study investigated the effect of alcoholic beverages on the parameters of oxidative stress in the liver of adolescent male rats. A number of studies have been conducted in animal models to analyze the influence of alcohol overconsumption on the hepatic damage during adolescence [27], or the damage occurring in progeny due to alcohol consumption by the mother [28]. However, most of the studies focusing on adolescent organisms considered only binge drinking, and not prolonged drinking, as it was justified that binge drinking is the most common way of ethanol intake among adolescents [27]. However, there is an increasing number of adolescents abusing alcohol and addicted to alcohol, which results also in an increasing number of young adults with ALD. Taking this into account, in the presented study, the results were compared with the results of studies with prolonged alcohol exposition (from 3 weeks to 3 months). Moreover, the present study included an assessment of various alcoholic beverages, which was not performed in the previous studies of other authors, as in a majority of studies using animal models, the influence of overconsumption of alcohol is analyzed only for ethanol.

ADH is a key enzyme that participates in the aerobic metabolism of ethanol [14]. However, in the present study, the ADH activity of the studied young animals did not differ from that of the control animals when various alcoholic beverages were applied. Similar results were reported in the study by Yamasaki et al. [29], in which the Lieber–DeCarli diet was given for 5 weeks to mature rats. By contrast, Tahir and Sultana [30] stated that mature rats exhibited a higher ADH activity after 4 weeks of increasing consumption of ethanol (5, 8, 10, and 12 g per kg of body mass) compared with the control animals, while Chandrasekaran et al. [31] stated an increased in vitro ADH activity after prolonged ethanol consumption. The lack of significant differences between the experimental groups and control group, as observed in the present study, may be attributed to the ethanol concentration of 10% in the applied alcoholic beverages, which may have not been enough to increase the ADH activity significantly, or the amount of alcohol consumed may have been adequately converted by ADH, and so, the metabolic pathway of ethanol detoxification was not disturbed. It may be also believed that while ethanol is consumed by young animals, some adaptive mechanisms of ethanol detoxification are activated, but this hypothesis requires verification by further studies.

When ethanol is consumed continuously, or at a high concentration, by an organism, the function of ADH is intercepted by CYP2E1, as Yamasaki et al. [29] noted an increase in the expression of CYP2E1 protein in the liver of mature rats that received the Lieber–DeCarli diet. In the present study, a significantly higher level of CYP2E1 in comparison with the control group was observed only in the animals that received beer for 6 weeks, but not in those receiving other alcoholic beverages. This finding indicated that the activity of the MEOS system was increased only when this alcoholic beverage was consumed. Similar to the present study, a lack of significant influence by ethanol and wine was also stated in the study by Hidestrand et al. [32], while no differences in the CYP2E1 activity in the liver were observed in mature male Sprague-Dawley rats that intragastrically received a diet with light or dark beer (5.8 g of ethanol per kg of body mass), which was attributed by the authors to the fact that the amount of ethanol was insufficient to induce the MEOS system. In addition, as stated in the present study for beer, a significant influence may be attributed to the composition of beer as a complex food product (compared with ethanol solution), as a lot of substances in beer may potentially interact with the CYP system, and such interactions may be of two different types. Flavonoids that are typical for beer, for example, xanthohumol, isoxanthohumol, and 8-prenylnaringenin, may in vitro inhibit CYP2E1, but polycyclic aromatic hydrocarbons may induce the activity of cytochrome P450 [33]. Thus, it may be speculated that growing organisms may be more prone to the potentially unfavorable components of beer, which may be due to the increase in the level of CYP2E1.

The liver metabolizes alcohol by ADH and cytochrome P450 2E1 (CYP2E1), while both the process of metabolism and the resulting metabolites induce the production of ROS, which intensify oxidative stress [34]. However, in the present study, no differences in the level of TBARS were found in the liver of the studied groups compared with the control animals. Similarly, in a study by Rodrigo et al. [35], no differences in TBARS levels were observed in mature Wistar rats that received either wine or ethanol solution (12.5% of ethanol) for a month, compared with the control group. According to the authors, this was associated with the antioxidative properties of the polyphenolic compounds, which may prevent the oxidation of the low-density lipoprotein (LDL) and the resultant peroxidative changes in cells.

Numerous studies in rats or mice have proved the intensified oxidation of lipids, which was assessed based on the level of TBARS, after the administration of ethanol at a concentration of 18–30% [36,37,38,39,40,41], but they also showed an increased level of protein carbonyl groups in the liver as a result of the intensification of oxidative stress by ethanol consumption [40,41,42]. According to some authors, the process of protein oxidation may be of great importance in the pathogenesis of ALD [41], as well as in the pathogenesis of the non-alcoholic fatty liver disease (NAFLD), including non-alcoholic steato-hepatitis (NASH) [43]. In the present study, the highest level of protein carbonyl groups observed in the liver was attributed to beer consumption, which may once again suggest the adverse effect of some components present in beer, or the higher vulnerability of growing organisms to those components. So far, no studies have been published assessing the influence of specific components of beer on the process of protein oxidation, so it would be valuable to specify which polyphenolic compounds of beer may be responsible for this activity. However, some suggestions can be formulated based on the in vitro study by Carvalho et al. [44], which showed that lower doses of xanthohumol protected the DNA of yeast cells against oxidative damage, whereas higher doses exerted a pro-oxidative effect. Therefore, it may be concluded that xanthohumol may have caused the pro-oxidative action that was observed after prolonged consumption of beer.

Some studies suggest that moderate consumption of fermented alcoholic beverages, such as beer, may reduce the development of liver steatosis to some extent [45]. In the study by Hege et al. [46], it was found that iso-alpha-acids present in the extract of hops significantly reduced the progression of the acute liver damage caused by ethanol intoxication in mice. This may be interpreted as a protective effect of the iso-alpha-acids, but it is not known if the same effect may be observed after prolonged exposure, as for beer in the present study.

An important observation of the present study was the higher level of protein carbonyl groups in the group of rats that received wine, compared with the control group. However, this finding was not confirmed by the studies of other authors. For instance, a study showed that in miniswine receiving an atherogenic diet and red wine or ethanol for 4 weeks, the protein oxidation was lower compared with the control group [47]. As indicated by Pandey and Rizvi [48], resveratrol present in red wine protects proteins against oxidation, but the effect is dose-dependent. On the other hand, resveratrol may also be a pro-oxidative compound [49]. Thus, it may be understood that in growing organisms, the possibility of observing such an influence may be higher, and hence, the higher level of protein carbonyl groups may be attributed to the influence of resveratrol.

Ethanol may increase the activity of liver enzymes in mature organisms [50,51,52], but in the present study, the lowest activity was found in the group of animals receiving beer compared with the other groups. Therefore, it may be assumed that any component of beer may have simultaneously influenced the level of protein carbonyl groups and the activity of ALT in the liver of growing rats, but this should be confirmed by further studies. According to Apte et al. [53], a decreased activity of liver enzymes may indicate the intensified restorative and repairing processes in hepatocytes. In the present study, ethanol was administrated from the 30th day of life, and hence, it may be hypothesized that in the case of very young animals, the regenerative potential is higher than for the mature ones. This is also confirmed by the study of Biondo-Simões et al. in Wistar rats [54], which proved that regenerative potential depends on age and it is higher in younger animals than for older ones, and other authors also suggest that regenerative potential decreases with age [55,56,57].

While summarizing the obtained results, it should be indicated that maturation is a process that is associated not only with visible physical changes in the body but also with several metabolic and hormonal changes. As suggested by Pérez-Navero et al. [58], oxidative stress may be associated with the maturation process itself, as an increased level of lipid peroxidation products was observed in healthy boys during adolescence compared with younger ones, before maturation process, indicating the role of lipid peroxidation in the development of oxidative stress during the adolescence period. The role of oxidative stress in various diseases occurring in growing adolescents is the subject of many studies [59,60,61], but so far little is known about the oxidative stress occurring in healthy children and adolescents; therefore, it may be necessary to assess the parameters of this process in healthy adolescents during the process of maturation. Most importantly, if alcohol consumption during adolescence is a significant predictor of its excessive consumption in adulthood [62], and if it is associated with oxidative stress and possibly the risk of liver damage and ALD, then this serious public health issue requires further studies.

## 5. Conclusions

In the present study, the animals receiving beer for 6 weeks showed increased levels of CYP2E1 and protein carbonyl groups, as well as decreased activity of ALT, compared with the control group. The results suggested that some components of beer, other than ethanol, are responsible for its influence on the markers of oxidative stress and liver inflammation observed in the animal model of prolonged alcohol consumption in adolescents. Taking this into account, beer consumption in adolescents, which is a serious public health issue, should be assessed in further studies to broaden the knowledge of the progression of liver damage caused by alcohol consumption in this group.

## Figures and Tables

**Table 1:** The acclimatization and experimental procedures.

Time		20th Day	21th–30th Day	From 31st Day, for Six Weeks
Group	Control	Animals divided into groups	Acclimatization procedure with no alcoholic beverage	Experiment with no alcoholic beverage
	Experimental (ethanol)		Acclimatization procedure with alcoholic beverage: —21st–22nd day—2% alc. vol. —23rd–24th day—4% alc. vol. —25th–26th day—6% alc. vol. —27th–28th day—8% alc. vol. —29th–30th day—10% alc. vol.	Experiment with alcoholic beverage (10% alc. vol.)
	Experimental (beer)			
	Experimental (red wine)			
